# Fatal Transdermal Fentanyl Patch Overdose in a Child

**DOI:** 10.7759/cureus.6755

**Published:** 2020-01-23

**Authors:** Mark A Hilado, Ariana Getz, Rachel Rosenthal, Daniel D Im

**Affiliations:** 1 Pediatrics, University of Southern California Keck School of Medicine, Los Angeles, USA

**Keywords:** overdose, fatal, fentanyl, patch

## Abstract

A previously healthy three-year-old girl was brought into the emergency department by ambulance after being found unresponsive with a family member’s fentanyl patch found adherent to her lower back. A head CT scan showed global cerebral edema and the patient progressed to brain death. An initial standard urine drug screen was negative for opiates, however, subsequent specific urine assay testing was found to be positive for fentanyl and norfentanyl. This case highlights the dangers of not properly disposing of used fentanyl patches as they may still contain enough fentanyl to cause respiratory failure and subsequent unintentional death in children. Prescribing physicians and pediatricians should advise care providers that fentanyl patches should be carefully stored, monitored, and disposed of properly in order to prevent accidental exposure to the pediatric population. Furthermore, synthetic opiates such as fentanyl will not read as positive on routine urine drug screens and will require specific urine assays.

## Introduction

Transdermal fentanyl is now commonly used for chronic pain management in oncologic patients. The advantages of transdermal delivery of fentanyl include avoidance of hepatic first passage metabolism, low enteric absorption, and the ability to bypass oral intolerance or non-compliance [1]. Fentanyl delivered via the transdermal patch is deposited in the keratinaceous layer of the epidermis, which allows for prolonged and continuous administration. Typically, the longevity of a single fentanyl patch is 72 hours. It is designed to facilitate consistent and continuous diffusion of fentanyl over the given time period. Previously worn fentanyl patches may retain up to 28-84% of the initial potency of the drug.

While there is limited information on younger pediatric populations, older pediatric patients have similar pharmacokinetics to that of adults for the rate of fentanyl delivery. The mean time to maximal serum concentrations has been found to be 36 hours. One study found that 1.5 to 5-year-old patients had fentanyl plasma concentrations almost twice as high compared to that of adult patients. With younger patients, other effects such as variability in skin temperature and thickness must be taken into account with regard to the delivery and concentration of fentanyl. Skin temperature elevation can lead to a gradual 10- to 15-fold increase in cutaneous blood flow, enhancing the absorption of the transdermal fentanyl [2].

## Case presentation

**Figure 1:**
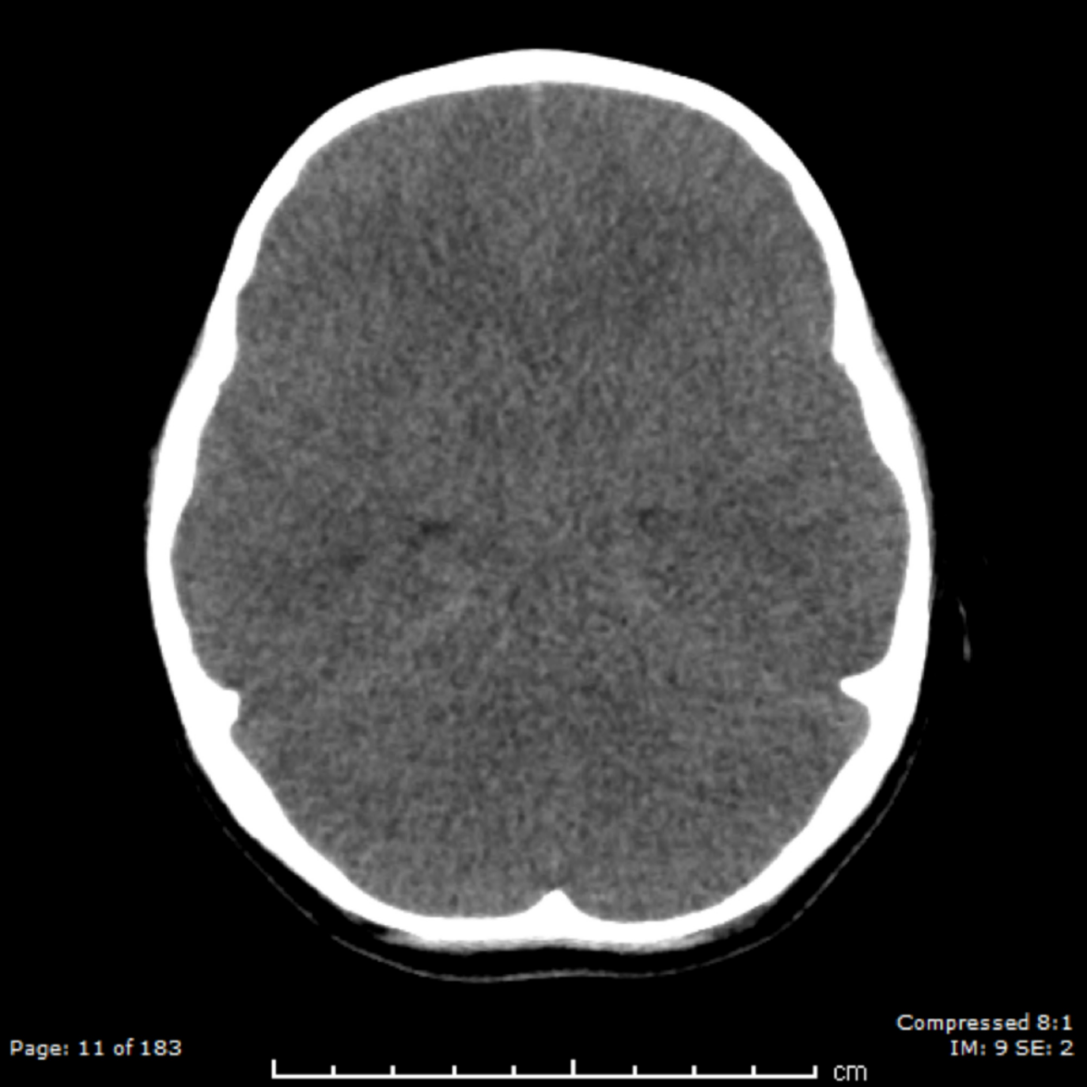
Head CT showing global cerebral edema

A 3-year-old girl with no significant past medical history was brought into the emergency department by ambulance after being found unresponsive at home. She had reportedly awoken early that morning requesting to sleep with her grandmother. When the grandmother had returned a few hours later, she had found that the child was still sleeping and had likely been asleep for approximately five hours. The child’s lips had reportedly turned blue, and she had not been breathing. A fentanyl patch had been found attached to the child’s lower back. The grandmother had been using 75 mcg fentanyl patches for pain management, and she had reported episodes of becoming sweaty at night and having trouble keeping the patch adhered in position, often needing to move it to another location on her body. Upon finding the child unresponsive, the grandmother had called emergency medical services (EMS) and begun chest compressions. The EMS report noted palpable pulses and shallow, slow respirations, with oxygen saturation of 80%, which improved with bag-mask ventilation. The estimated weight of the child was 15 kg, and she had been noted to have pinpoint pupils bilaterally. She had been given 1.5 mg intramuscular (IM) naloxone in the field, and an additional 1.5 mg intravenous (IV) naloxone en route, with no improvement. On arrival to the emergency department, the patient continued to be unresponsive with a Glasgow coma scale (GCS) score of 3, agonal respirations, and hypotension. Her pupil size was noted to be 2 mm in diameter and reactive bilaterally. A third dose of naloxone 1.5 mg IV was administered with no improvement. Resuscitation included ceftriaxone and vancomycin administration for sepsis, a dose of hypertonic saline (3%) for suspected elevated intracranial pressure, and normal saline boluses for hypotension. A norepinephrine infusion was initiated for sustained hypotension. No narcotics or sedative medications were given. She continued to be unresponsive and required intubation. A head CT scan was done, which revealed global cerebral edema (Figure 1). The patient was shifted to the pediatric intensive care unit (PICU).

On arrival to the PICU, the patient remained intubated with a GCS score of 3, and her pupils were noted to be dilated to 5 mm and nonreactive. A fundoscopic exam revealed bilateral papilledema. The remainder of the neurologic exam was notable for no response to maximum stimulation in all four extremities, no cough or gag reflex, and absent corneal reflex. She had no outward signs of trauma, no bruising or bleeding, and was found to have an area of adhesive residue on her lower back where the grandmother reported finding the fentanyl patch adherent to her skin.

The lab results of the patient are listed below in Table 1. An initial standard urine drug screen was negative for amphetamines, barbiturates, benzodiazepines, cocaine, opiates, phencyclidine, and oxycodone. Serum ethanol, acetaminophen, and salicylate levels were undetectable as well. Neurosurgery was consulted and, given the concern for brain death based on physical examination and imaging, no surgical intervention was indicated. An electroencephalogram (EEG) showed no evidence of seizure activity but did show a severely attenuated background with very minimal brain wave activity. Echocardiogram was completed without evidence of cardiac etiology for respiratory failure. The patient was continued on medical therapies to maintain hemodynamic stability, treat any possible underlying infectious etiologies, and correct electrolyte disturbances. She showed no improvement after 48 hours of medical treatment. She remained unresponsive with a GCS of 3 and persistent absence of brainstem reflexes on examination. No infectious, cardiac, or neurologic etiologies were found. A non-accidental trauma workup including skeletal survey and ophthalmologic examination was negative. Subsequent urine testing specifically for fentanyl was sent on day two of admission and found to be positive for fentanyl (2.7 ng/mL; cutoff: 0.5 ng/mL) and norfentanyl (48.8 ng/mL; cutoff: 0.5 ng/mL). The patient had not received any fentanyl from EMS, in the emergency department, or while admitted to the PICU. On day three, the patient underwent a first brain-death examination, which revealed absent cortical activity and absent brain stem reflexes consistent with brain death. A second brain-death exam was completed on day four, which confirmed brain death. The patient was declared dead by brain death criteria secondary to a fentanyl overdose.

**Table 1:** Biochemical testing results

Test	Patient's value	Reference range
Sodium (mmol/L)	150	135-145
Potassium (mmol/L)	3.4	3.6-5.2
Chloride (mmol/L)	124	100-108
Bicarbonate (mmol/L)	13	22-29
Blood urea nitrogen (mg/dL)	23	8.0-24
Creatinine (mg/dL)	1.08	0.8-1.3
Calcium (mg/dL)	8.1	8.6-10
Phosphorus (mg/dL)	3.8	2.5-4.5
Magnesium (mg/dL)	2.3	1.3-2.4
Alanine aminotransferase (U/L)	131	0-40
Aspartate aminotransferase (U/L)	846	8-48
Albumin (g/dL)	3.9	3.5-5.0
Direct bilirubin (mg/dL)	<0.2	0.1
Total bilirubin (mg/dL)	<0.2	0.6

## Discussion

Fentanyl is a synthetic opioid that was first synthesized in 1960 by Paul Janssen. It resulted from an effort to improve the potency and safety profile of morphine, particularly with minimal cardiovascular effects. Today, alternative administration routes of fentanyl including lozenges, sublingual patches, sublingual sprays, intranasal sprays, and transdermal patches have become important forms of treatment for patients with chronic pain, especially for patients with cancer [2]. In comparison to other opiates such as morphine, fentanyl is a much stronger µ-receptor agonist, and it can have up to 75-100 times the potency of morphine. The metabolites of fentanyl vary from morphine in that they cannot be detected by the monoclonal antibodies used in basic urine drug screens [3-7]. In our case, the patient's grandmother had been using a standard 75 mcg fentanyl patch, which actually delivered 75 mcg/hr of fentanyl. Normally, a full patch contains 12.6 mg of fentanyl and this dosing gets administered over a period of seven days. In a patient who weighs 15 kg, such as in our case, this dose is equivalent to running a fentanyl infusion of 5 mcg/kg/hr while bypassing some hepatic first-pass metabolism. This case emphasizes the importance of anticipatory guidance and precautionary care with regards to transdermal pain management safety. In addition, it is imperative to remind physicians of the limitations of standard urine drug screens. One of the initial laboratory tests sent for the workup of this patient was a urine toxicology screen. The initial screen returned negative for opiates. Without a reliable history, this negative result could have altered the differential diagnosis. While opiates are stated to be found on urine drug screens, this is limited to morphine and codeine, as these are naturally occurring opiates and opiate metabolites. Specific testing for fentanyl and norfentanyl must be ordered and sent separately from the typical urine toxicology test. Norfentanyl is the major metabolite of fentanyl with up to 26-55% being eliminated in the urine [8].

It is a common practice for prescribing providers and pediatricians to discuss with patients and the patients' caregivers the importance of keeping medications out of the reach of children; however, transdermal patches are often overlooked. An FDA report reviewing cases between 1990 (the year the first fentanyl patch received approval) and 2012, identified 30 cases of accidental pediatric exposure. In these cases, children had encountered patches that had either been loosely attached, fallen off, or improperly stored/discarded. Of the 30 cases, 10 led to death, and 19 were patients two years of age or younger. As of September 2013, the FDA began requiring colored patches to increase visibility. One study looked at 1,917 pediatric transdermal drug delivery systems over 10 years from 2006 to 2015 and found that exposures involving fentanyl resulted in higher rates of major or moderate medical outcomes and were associated with multiple deaths [9]. Toddlers continue to be at the highest risk for mistaking used patches for stickers, pretend tattoos, or bandages. The FDA advises that patients wearing a fentanyl patch add an additional adhesive over the patch and administer patch checks multiple times per day. Regarding patch disposal, the FDA has placed fentanyl patches on its "flush list" and advises folding them in half and flushing them down the toilet after use. However, it is ideal to hand them over to a drug take-back site where they can be properly discarded [10].

## Conclusions

It is important for physicians to be aware that synthetic opiates such as fentanyl are not routinely tested positive on standard urine drug toxicology screening and require specific urine assays to assess levels. Also, used fentanyl patches may still contain enough fentanyl to cause respiratory failure and subsequent death in a pediatric patient. Finally, fentanyl patches should be carefully stored, monitored, and disposed of properly in order to prevent accidental exposure to the pediatric population.
